# Evaluation of Serum Soluble Lectin-like Oxidised Low-Density Lipoprotein Receptor-1 (sLOX-1) Level in Children with Non-Complicated Type-1 Diabetes Mellitus (T1DM) and Its Relationship with Carotid Intima Media Thickness (cIMT)

**DOI:** 10.3390/jcm14030935

**Published:** 2025-01-31

**Authors:** Sukriye Ozde, Fatma Yavuzyilmaz, Mehmet Ali Ozel, Osman Kayapinar, Cem Ozde, Gulsah Akture, Ilknur Arslanoglu

**Affiliations:** 1 Department of General Pediatric, Faculty of Medicine, Duzce University, 81620 Duzce, Turkey; sukriyeozde@gmail.com; 2 Department of Pediatric Endocrinology, Faculty of Medicine, Duzce University, 81620 Duzce, Turkey; fatma_yavuzyilmaz@hotmail.com (F.Y.); ilkilkars@yahoo.com (I.A.); 3 Department of Radiology, Faculty of Medicine, Duzce University, 81620 Duzce, Turkey; drmaliozel@gmail.com; 4 Department of Cardiology, Faculty of Medicine, Duzce University, 81620 Duzce, Turkey; drcemozde@gmail.com (C.O.); gulsahkrtls@hotmail.com (G.A.)

**Keywords:** type-1 diabetes mellitus (T1DM), soluble lectin-like oxidized low-density lipoprotein receptor 1 (sLOX-1)

## Abstract

**Background**: The objective of this study was to evaluate serum soluble lectin-like oxidized low-density lipoprotein receptor-1 (sLOX-1) levels in children with type-1 diabetes mellitus (T1DM) without any atherosclerotic complications and to investigate whether there was an association with early atherosclerotic processes in these children. **Methods**: The study’s design entailed a prospective cross-sectional observational study methodology. The patient group consisted of 80 consecutive children aged 8–18 years who had been diagnosed with T1DM for at least ten years and had not developed any chronic clinical complications related to T1DM. The control group consisted of 72 completely healthy children with similar demographic characteristics. Serum levels of sLOX-1 were measured, and carotid intima-media thickness (cIMT) was evaluated using ultrasonography in all subjects. **Results**: A statistical analysis of the results was conducted. The serum sLOX-1 level was found to be significantly higher in the patient group than in the control group (0.49 ± 0.11 vs. 0.82 ± 0.35; *p* < 0.001). The statistical significance observed was maintained in the multivariable logistic regression analysis (*p* < 0.001). A significant correlation was identified between cIMT and serum sLOX-1 levels (r = 0.669, *p* < 0.001). The receiver operating characteristic curve for sLOX-1 indicated that a cutoff value greater than 0.65 ng/mL was associated with T1DM. **Conclusions**: Serum sLOX-1 levels were markedly elevated in children with T1DM who had not yet manifested chronic complications. These findings suggest that elevated serum sLOX-1 levels may be associated with the progression of atherosclerosis in children with T1DM.

## 1. Introduction

Atherosclerotic cardiovascular disease (ACVD) represents the leading cause of mortality and morbidity in individuals with type 1 diabetes mellitus (T1DM), with this population exhibiting an elevated incidence of ACVD in comparison to those without diabetes [1,2,3]. Individuals with early-onset T1DM are approximately 30 times more likely to experience severe adverse cardiovascular outcomes than healthy individuals. Individuals who receive a diagnosis of T1DM before reaching the age of ten exhibit an average life expectancy that is sixteen years shorter than the general population. Those who receive a diagnosis between the ages of 26 and 30 have a life expectancy that is ten years shorter than average [3]. Patients diagnosed with type 1 diabetes have been observed to undergo an accelerated atherosclerotic process, starting with early endothelial impairment during childhood [4].

Non-invasive ultrasonographic measurement of carotid intima-media thickness (cIMT) is used in clinical practice to evaluate subclinical atherosclerosis. Endothelial dysfunction is a well-known indicator of the subclinical phase of atherosclerotic progression, characterised by intima-media thickening in the entire arterial system [5]. cIMT is directly related to metabolic regulation in diabetes mellitus. As diabetic exposure progresses, cIMT increases progressively and serves as a predictor of atherosclerotic macro- and microvascular complications. Furthermore, increased cIMT has been reported in pediatric T1DM subjects in comparison to healthy controls [6,7]. Despite the paucity of data on the atherosclerotic processes associated with T1DM in children, studies have indicated the presence of early endothelial damage and the progression of atherosclerosis, as evidenced by the increase in cIMT observed in the initial years following the onset of atherosclerosis [8].

The pathogenesis of atherosclerosis is not yet fully understood, as it is a complex process. It is believed that high serum levels of low-density lipoprotein (LDL) play a key role in the initiation and progression of the atherosclerotic process [9]. Atherosclerosis is caused by several mechanisms, including lipid accumulation in the intima and media of arteries, particularly oxidized cholesterol particles, low-intensity chronic inflammation, protein accumulation in the extracellular matrix, vascular calcification, and endothelial damage [10]. There has been a notable increase in interest in the role of high levels of oxidized cholesterol and its derivatives in atherosclerosis over the past decade.

The lectin-like oxidized low-density lipoprotein receptor-1 (LOX-1) is considered a significant trans-membrane scavenger receptor for oxidized low-density lipoprotein (ox-LDL) in endothelial cells. However, it is also observed to be expressed in a variety of other cell types, including macrophages, smooth muscle cells, and fibroblasts [11]. The LOX-1 has been implicated in the initiation and progression of atherosclerosis [11]. Under normal conditions, LOX-1 expression is quite low. Nevertheless, it is upregulated in related cell lines in response to proatherogenic factors, including ox-LDL, glucose and glycation end products, pro-inflammatory and pro-oxidative conditions, and shear stress [12]. The upregulation and activation of LOX-1 activates pro-inflammatory processes. The activation of matrix metalloproteinases and cell adhesion molecules, in addition to the initiation of apoptotic pathways, has been observed. All these processes increase endothelial dysfunction, atherosclerotic plaque formation and progression, and atherosclerotic plaque vulnerability [13].

sLOX-1, the soluble form of LOX-1, is present in the circulation as a result of enzymatic cleavage promoted by the pro-inflammatory extracellular environment [11]. The efficacy of diagnostic and prognostic assessments based on the evaluation of serum slox-1 levels in adults has been investigated in clinical studies. In this context, slox-1 has emerged as a promising biomarker for the detection of atherosclerotic etiology in several diseases, including coronary artery disease, aortic dissection, and ischemic stroke [11]. Furthermore, it may facilitate the prediction of atherosclerotic diseases, the classification of atherosclerotic disease severity, the assessment of atherosclerotic burden, and the monitoring of treatment response [11,14]. However, the existing literature contains an insufficient amount of data concerning the relationship of serum sLOX-1 levels with subjects predisposed to atherosclerosis and subclinical atherosclerosis. To the best of our knowledge, no evaluation of serum sLOX-1 levels in patients with T1DM in childhood has yet been conducted. In this study, therefore, the investigation focused on serum slox-1 levels in children diagnosed with T1DM and exhibiting no clinical atherosclerotic complications. The relationship between these levels and cIMT, a marker of subclinical atherosclerosis in these patients, was also examined.

## 2. Materials and Methods

### 2.1. Study Population

The study was approved by the Ethics Committee of Düzce University Faculty of Medicine. All subjects and/or their parents/legal guardians provided written informed consent for participation. All authors have declared in writing that they adhere to the principles of the World Medical Association Declaration of Helsinki regarding the ethical conduct of research involving human subjects. The present study was carried out as a prospective cross-sectional observational study. This study was conducted at the Department of Pediatrics at the Düzce University Medical Faculty Training and Research Hospital, which is a tertiary health care institution. The study included 100 children who were followed up in our clinic, had been diagnosed with T1DM for a minimum of 10 years, had no chronic clinical complications related to T1DM, and were younger than 18 years of age. The control group comprised 100 healthy children with similar demographic characteristics to the patient group who presented themselves at the Healthy Child Outpatient Clinic. In this context, subjects who were younger than 18 years of age who did not have a known history of chronic disease and a regular medical treatment used accordingly, who did not have any acute disease in the last 10 days with medical treatment used for this reason and vaccination in the last 1 month, and who also applied to the “Healthy Child Outpatient Clinic” were selected for general control.

Individuals with any clinical chronic complication of T1DM, obesity, overweight, or a history of endocrine disorders other than T1DM, a history of autoimmune and rheumatological diseases, a history of chronic allergic diseases, a history of neoplasia, a history of fever of unknown cause at any time, or a history of infectious diseases in the last four weeks were all excluded from the study. Furthermore, individuals with a history of COVID-19-related multisystem inflammatory syndrome in children (MIS-C) at any time, a history of steroid, cytotoxic agent, antihypertensive therapy, or lipid-lowering drug use at any time, and a history of non-steroidal anti-inflammatory drug use in the last four weeks were all excluded from the study. Furthermore, subjects who did not participate in scheduled pediatric endocrinology outpatient clinic visits every three months and those with incomplete or inconsistent data recorded in the electronic health record system were also excluded from the study.

The diagnosis of T1DM was made in accordance with the criteria set out in the International Society for Pediatric and Adolescent Diabetes (ISPAD) consensus report [15]. All T1DM patients in the present study were children and adolescents with T1DM who had been followed up in our clinic from the time of diagnosis until the time of the present study, who had regular follow-up visits every three months, and those who had been followed up in this way for at least 10 years.

A detailed medical history was obtained for each patient included in the study, and a physical examination was performed. Demographic and clinical characteristics, anthropometric values, pubertal status, a history of acute and chronic complications related to diabetes, insulin treatment regimens, and metabolic control status were obtained from the Pediatric Endocrinology Clinic’s registered file system. In addition, the standard deviation values for height, weight, and body mass index (BMI) were evaluated in accordance with the standards of the local community. The pubertal status of each case was determined in accordance with the Tanner criteria. The micro- and macrovascular complications of diabetes and the metabolic control status of the subjects were determined based on the ISPAD guidelines [16,17].

All children with T1DM included in the study are subject to regular follow-up at three-month intervals by our clinic. All clinical and laboratory information obtained during follow-up is recorded manually in patient files and electronically in the hospital system. Hence, all data obtained between the time of initial diagnosis and start of the study are accessible for review in the existing records. The HbA1c values for each patient were obtained from their records and a mean HbA1c level was calculated to encompass all values recorded from diagnosis until the start of the study.

### 2.2. Measurement of Carotid Intima-Media Thickness

An experienced radiologist examined cIMT using ultrasound. They were blinded to the subjects’ characteristics. The examinations took place in a quiet room at a constant temperature following an overnight fast. Images were captured with the subjects in a supine position and their necks extended, using a standard magnification of approximately 10 mm to focus on the posterior wall distal to the left and right common carotid arteries, just proximal to the carotid bulb. Images were only obtained when both the anterior and posterior edges of the arterial walls were clearly visible, ensuring proper alignment at a right angle to the vessel. The most optimal images for both carotid arteries were selected and recorded. We measured the cIMT of all subjects using a GE Logiq S8 ultrasound device (2018; General Electric Healthcare, Chicago, IL, USA), with a 6–10 MHz linear array transducer and electrocardiogram gating during diastole. A clinical radiologist analyzed the images in a randomized manner. The intimal width was measured at three points in each carotid artery, and the mean width was calculated. The average of the right and left cIMT measurements was recorded [18]. Measurements were repeated for 15 patients from the study group to assess variability. The reproducibility of the ultrasound-derived cIMT was evaluated. This was determined by dividing the standard deviation of the differences between repeated measurements by the mean of the repeated measurements and expressing it as a percentage. The intra-observer variability was found to be less than five percent.

### 2.3. Blood Sampling and sLOX-1 Measurements

A total of 6 mL of blood was collected from each subject participating in the study. The samples were obtained from the antecubital vein after an overnight fast and were placed in both plain and EDTA bottles. The serum was separated into Eppendorf Safe-Lock tubes by centrifugation at 2000 rpm for 10 min and stored at −80 °C until the time of measurement. Following the collection of samples from all subjects, the serum sLOX-1 level was quantified using a commercially available enzyme-linked immunosorbent assay (ELISA) kit, in accordance with the manufacturer’s instructions (Aviscera Bioscience, Santa Clara, CA, USA) [19]. The ELISA was employed to analyze all samples in duplicate, with the results averaged to minimize potential errors in measurement and calculate the final concentration in the samples. The intra-assay coefficient of variability was found to be less than 10%, while the inter-assay coefficient of variability was determined to be less than 15 percent. The sLOX-1 level was measured in ng/mL. The lower limit of detection for sLOX-1 was established at 0.50 ng/mL. The measurement of fasting blood glucose, total cholesterol, high-density lipoprotein (HDL), triglycerides, low-density lipoprotein (LDL), sodium, and potassium were conducted by means of an autoanalyzer (Abbott Architect C 16000, USA). The hexokinase method, the enzymatic method, the accelerator selective detergent method, the glycerol phosphate oxidase method, and the Friedewald formula were used in the process, respectively. The measurement of serum sodium and potassium concentrations was conducted using an indirect ion-selective electrode method, employing the same auto-analyzer. Serum TSH concentrations were measured using a chemiluminescent immunoassay method. A complete blood count was conducted with an automated haematology analyzer (Beckman Coulter, Brea, CA, USA) within one hour of the venipuncture. Furthermore, the quantification of urea, creatinine, and uric acid was conducted using an autoanalyzer (Abbott Architect 16200, Lake Forest, IL, USA) using the spectrophotometric method. Glomerular filtration rate (GFR) was calculated using the Schwartz equation to evaluate renal function. The measurement of high-sensitive C-reactive protein (hs-CRP) was undertaken with an analyser (Siemens BN II, Forchheim, Germany) using the nephelometric method. The HbA1c levels were determined by high-performance liquid chromatography using a Primus Ultra 2 analyzer (Primus Corporation, Kansas City, KS, USA).

### 2.4. Statistical Analyses

The mean, standard deviation, median, minimum, maximum value, frequency, and percentage were used for the purpose of descriptive statistics. The distribution of variables was measured using the Kolmogorov-Smirnov and Shapiro-Wilk tests. Independent samples *t*-tests were used to analyze independent quantitative data with a normal distribution. Mann-Whitney U tests were used in the analysis of independent quantitative data with non-normal distribution. The Chi-Square test was used for the comparison of the qualitative data. ROC analysis was used to demonstrate the effect level. The investigation of the effect level was conducted through the use of univariate and multivariate logistic regression analyses. Spearman’s correlation analysis was employed for the purpose of correlation analysis. The statistical analyses were conducted using SPSS 26.0.

## 3. Results

A total of 152 subjects were included in the study, comprising 80 patients and 72 controls. Following a comprehensive evaluation of the demographic data of both groups, it was observed that there was no significant difference between the patient and control groups with regard to age, gender distribution, body surface area, body mass index, and blood pressure (*p* > 0.05). The results are presented in Table 1, which also provides a summary of the clinical features of the patient group.

**Figure 1:**
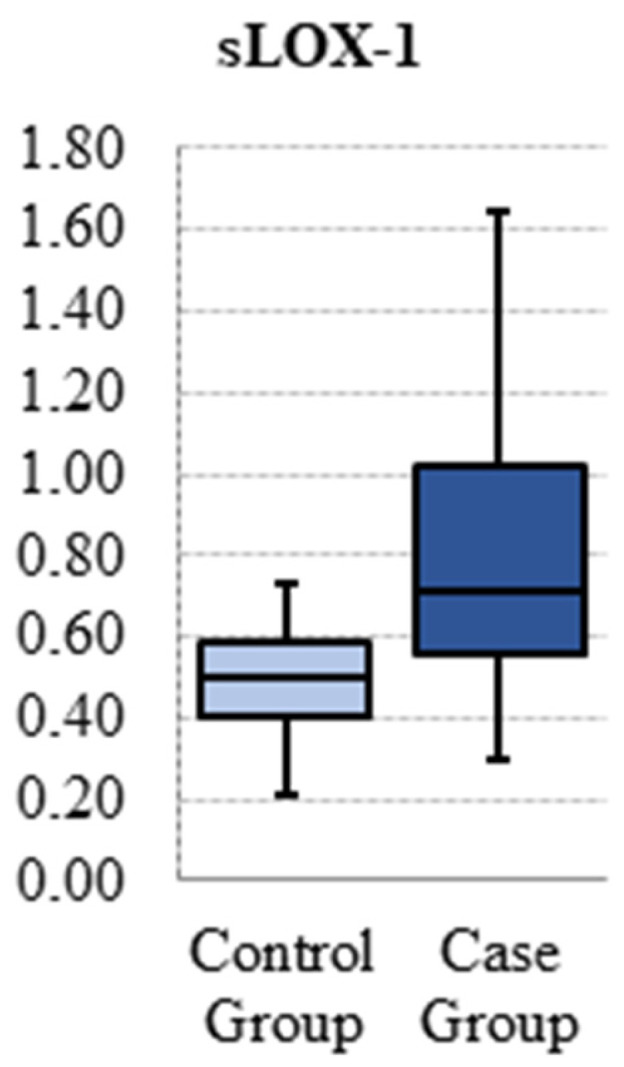
Plasma sLOX-1 Levels in Patient and Control Groups.

A comparison was made between the two groups in terms of their basic blood biochemistry parameters. The total cholesterol (145.5 ± 22.6 vs 166.1 ± 27.4; *p* < 0.001), HDL (48.3 ± 9.2 vs 58.2 ± 13.3; *p* < 0.001), hemoglobin (1 2.9 ± 1.3 vs 13.2 ± 1.1; *p* = 0.037), and high-sensitivity C-reactive protein (hs-CRP) (0.09 ± 0.12 vs 0.39 ± 0.26; *p* < 0.001) levels were found to be significantly higher in the patient group. The results are presented in Table 2. A comparison was made of the plasma sLOX-1 levels of the two groups, and it was determined that the plasma sLOX-1 levels were significantly higher in the patient group than in the control group (0.49 ± 0.11 vs 0.82 ± 0.35; *p*<0.001). The resulting data are presented in Figure 1. Furthermore, the cIMT was found to be significantly higher in the patient group than in the control group (0.35 ± 0.03 vs 0.47 ± 0.09; *p* < 0.001). The findings of these analyses are also presented in Table 2.

Multivariate logistic regression analysis was performed, with all parameters that were statistically significant being evaluated. The analysis demonstrated that only cIMT (Exp(B)/OR: 1.019, 95% CI: 1.006–1.032, *p*<0.001), HDL (Exp(B)/OR: 1.158, 95% CI: 1.070–1.254, *p*<0.001), hs-CRP (Exp(B)/OR: 0.775, 95% CI: 0.525–0.875, *p* = 0.003) and sLOX-1 (Exp(B)/OR: 1.670, 95% CI: 1.485–1.915, *p* < 0.001) were significantly associated with T1DM. The findings of the multivariate logistic regression analysis can be viewed in Table 3.

**Figure 2:**
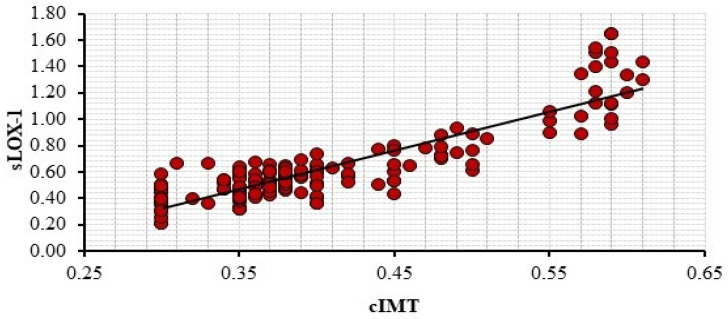
Correlation Analysis between Serum sLOX-1 Level and Carotid Intima-Media Thickness (cIMT).

The correlation between the variables identified as significant in the multivariant logistic regression analysis and mean-HgA1c with sLOX-1 was investigated. Only a significant correlation was found between cIMT and sLOX-1 (r = 0.669, *p*< 0.001). The results are presented in Table 4 and illustrated in Figure 2.

**Figure 3:**
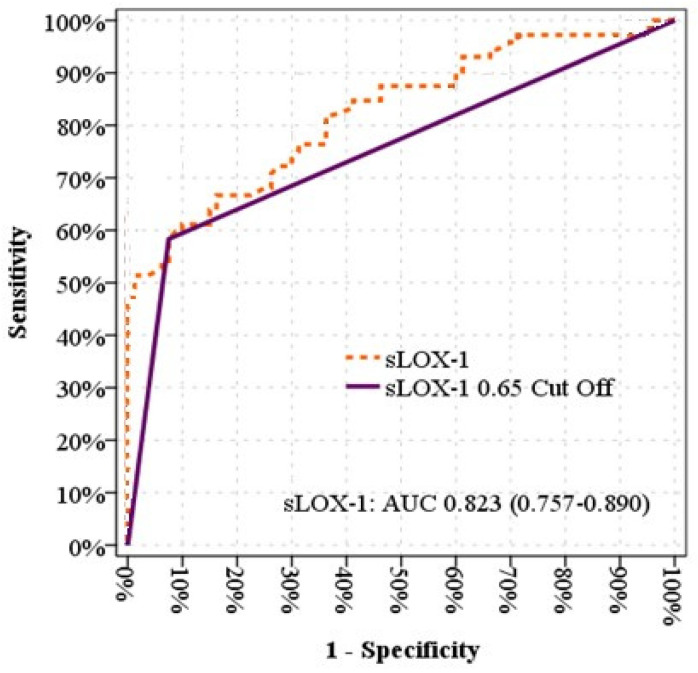
ROC curve.

As illustrated in Figure 3, a receiver operating characteristic (ROC) curve analysis was conducted in the patient and control groups to ascertain the sLOX-1 cutoff value for predicting patients with T1DM. The ROC curve for sLOX-1 demonstrated that a cutoff value greater than 0.65 ng/mL was associated with T1DM (58.3% sensitivity, 92.5% specificity, 87.5% positive predictive value, and 71.2% negative predictive value). The area under the curve was 0.754 (95% confidence interval: 0.674–0.835; *p* < 0.001).

## 4. Discussion

If we are correct, this appears to be the first study to investigate the serum sLOX-1 level in children with type 1 diabetes and evaluate its relationship with carotid intima-media thickness. The present study revealed that serum sLOX-1 levels, serum HDL levels, serum hs-CRP levels, and carotid intima-media thickness were found to be significantly increased in children with clinically uncomplicated T1DM when compared to the group of healthy controls. This significance was maintained in a multivariate logistic regression analysis. Furthermore, a significant correlation was identified between serum sLOX-1 levels and cIMT in children with T1DM.

The development of atherosclerosis in children with T1DM is the result of a complex interplay of genetic, environmental, and diabetes-specific factors. Hyperglycemia, a pathognomonic feature of T1DM, plays a crucial role in the initiation and acceleration of atherosclerosis [20]. Long-term exposure to hyperglycemia initiates a cascade of events, including increased oxidative stress, inflammation, and the formation of advanced glycation end products (AGEs). These processes result in the accumulation of lipids and inflammatory cells in the arterial wall, primarily endothelial dysfunction [20,21,22]. Endothelial dysfunction is one of the vascular complications of diabetes that occurs in the early stages of the disease and becomes systemic over time. This process occurs well before the onset of clinical macrovascular atherosclerotic complications associated with diabetes [20]. Studies have suggested that sLOX-1 may be a potential mediator of endothelial dysfunction. The association between sLOX-1 and endothelial dysfunction has been investigated in metabolic syndrome and polycystic ovary syndrome, which are known to cause increased insulin resistance and cardiovascular risk. Civelek et al. [23] reported that serum sLOX-1 levels were elevated in adults with metabolic syndrome, and this was associated with endothelial dysfunction. Oncul et al. [24] reported that serum sLOX-1 levels were increased in female subjects with polycystic ovary syndrome (PCOS) compared to healthy controls with similar demographic characteristics. They suggested that this result may be an indicator of endothelial dysfunction in women with PCOS. Stinson et al. reported that sLOX-1 levels are elevated in overweight/obese children and adolescents, particularly during and after puberty, and are associated with pro-inflammatory biomarkers and worsening cardiometabolic risk profiles. Furthermore, this study by Stinson et al. appears to be the only study in which sLOX-1 levels were assessed with a particular focus on children [25]. The present study demonstrated that slox-1 level was significantly elevated in children with non-complicated T1DM compared to healthy controls. This result may be associated with the subclinical progression of atherosclerosis in children with T1DM.

Several factors that may influence serum sLOX-1 levels in diabetic adults have been identified. Tan et al. demonstrated that hyperglycaemia and AGEs upregulate LOX-1 expression, which subsequently increases serum sLOX-1 levels [26]. Shiu et al. demonstrated that glycoxidized LDL, a degraded form of LDL that occurs due to diabetes, can induce LOX-1 expression in endothelial cells and may even be a stronger ligand than oxLDL [27]. In a study by Lam et al., the effect of systemic metalloprotease-10 (MMP-10) levels on the enzymatic cleavage of LOX-1 and the subsequent formation of sLOX-1 in adults with type 2 diabetes mellitus (T2DM) was investigated. The findings revealed that serum sLOX-1 levels in adults with T2DM were elevated in comparison to healthy controls and demonstrated a robust correlation with serum MMP-10 levels [28]. Elevated serum sLOX-1 levels have been observed in adults with T2DM and have been reported to be associated with an increased risk of diabetes-related macro-micro vascular complications. Indeed, sLOX-1 has been identified as a potential biomarker for several diabetic complications, including coronary artery disease [29], peripheral arterial disease [30], and nephropathy [31]. The current study suggested that serum sLOX-1 levels might be significantly higher in children with T1DM compared to healthy controls with similar demographic attributes. Most clinical studies investigating the relationship between diabetes and sLOX-1 have been conducted in adults and patients with T2DM. In this context, the present study appears to be the inaugural study on this subject, both in terms of its focus on T1DM and its exclusive focus on children and adolescents.

Carotid intima-media thickness has been proven to be an early marker of atherosclerotic progression and considered a robust predictor of future cardiovascular events. It has been demonstrated that cIMT can be employed in conjunction with established cardiovascular risk factors in a multitude of studies involving adults, thus enhancing cardiovascular risk profiles and formulating preventive strategies by identifying high-risk individuals [32]. cIMT is a non-invasive, reproducible, and relatively inexpensive method for the detection of preclinical atherosclerosis [18,32]. In a large-scale meta-analysis encompassing both adults and children, significantly increased cIMT values were observed in patients with T1DM in comparison to healthy controls [33]. Although most studies investigating cIMT levels in children and adolescents with T1DM employed a cross-sectional observational design, the findings from these studies consistently demonstrated that cIMT levels were increased in children with T1DM compared to healthy controls [34,35]. The present study revealed that cIMT may be significantly increased in children with T1DM, and that there may be a correlation between cIMT and serum sLOX-1 levels. Moreover, the absence of clinically significant macro- or microvascular atherosclerotic complications in the patients included in the study may be associated with the presence of subclinical atherosclerosis, as suggested by the observed correlation between increased serum sLOX-1 levels and cIMT.

The primary factors influencing the progression of atherosclerosis in children with T1DM are the duration of diabetes exposure and the level of glycemic control [20,21,22]. A longer duration of exposure to diabetes is associated with significant macro- and microvascular changes. In this context, an earlier onset of disease is an indicator of a poor prognosis. As in adults, poor glycemic control in children, as indicated by elevated HbA1c levels, is associated with increased carotid intima-media and aortic intima-media thickness, as well as arterial stiffness, which are important indicators of pre-atherosclerotic changes [36]. However, the present study did not identify a statistically significant correlation between mean-HgA1c levels and either cIMT or serum sLOX-1 levels. These findings may be attributed to the relatively short disease exposure duration and the fact that the mean HgA1c levels of the included children were relatively close to the good glycemic control range.

Several additional results, which did not form the focus of the present study but appear to be consistent with the data in the literature, were also obtained. Serum hs-CRP and HDL levels were found to be elevated in children diagnosed with non-complicated T1DM in contrast to levels observed in healthy control subjects. C-reactive protein (CRP) is an acute phase reactant synthesised predominantly within the liver. In preliminary studies, it was documented that the standard reference range for CRP in a healthy population without an acute disease state could reach up to 5 mg/L [37]. CRP has been demonstrated to play a pivotal role in the progression of atherosclerosis, from its initial stages. In vitro studies have demonstrated that CRP activates the complement system by binding to LDL, stimulates the release of tissue factor from macrophages, and thus may trigger thrombogenicity, forming a complex with enzymatically modified LDL particles, which are captured by macrophages and promote the formation of foam cells in atherosclerotic lesions [37,38]. CRP concentrations remain remarkably stable in healthy individuals, depending on the time of day and over extended periods ranging from days to months [39,40]. Especially when measured with highly sensitive (hs) assays that allow a reliable analysis even below the 5 mg/L limit, hs-CRP is an independent indicator of future atherosclerotic processes and is even predictive in clinically asymptomatic healthy young people [41,42]. In view of these findings, the present study was conducted with the objective of evaluating the subclinical early phases of atherosclerosis in children with T1DM using hs-CRP. Schalkwijk et al. reported that levels of the inflammatory marker hs-CRP were higher in patients with T1DM without macrovascular complications than in the control group [43]. Heier et al. found that hs-CRP levels were dependent on HbA1c levels and showed a positive correlation in diabetic children [44]. Another study by Weber et al. showed that increased hs-CRP was associated with HbA1c levels and indicated poor glycaemic control even in the very early stages of type 1 diabetes [45]. Recent studies have focused on T1DM cases in children and young age groups, and these have shown that poor glycemic control and dyslipidaemia are associated with high serum hs-CRP levels [46,47]. The results of the studies clearly demonstrate the presence of low-grade inflammation in T1DM even in childhood. It is widely accepted that HDL is a vasoprotective bioparticle with numerous anti-atherogenic and anti-inflammatory properties [48]. However, it has been reported that serum HDL levels may be comparatively elevated in individuals with type 1 diabetes mellitus, who demonstrate a higher risk of cardiovascular disease in comparison to the healthy population [49]. It has been hypothesised that HDL may transform into a dysfunctional pro-inflammatory bioparticle by losing its anti-atherogenic and anti-inflammatory properties. The process of this dysfunctional biotransformation is primarily influenced by systemic low-grade inflammation, and higher HDL levels in T1DM have been observed to be associated with this phenomenon [50].

It should be noted that this clinical study is subject to certain limitations. The study population is relatively small, and the study was designated as a cross-sectional observational study. Moreover, the children with T1DM included in the study were uncomplicated cases with a relatively short duration of disease exposure. Considering these considerations, it is not possible to definitively explain the causality of the results obtained. Ultrasonographic measurement of cIMT, which is widely accepted as a pre-atherosclerotic finding, is a semiquantitative method that is dependent on the operator.

In conclusion, the results of this investigation revealed that serum sLOX-1 level was markedly elevated in children with T1DM who had not yet manifested chronic complications when compared to healthy controls. Furthermore, a significant correlation was observed between serum sLOX-1 levels and cIMT in children with uncomplicated T1DM. It can be hypothesized that elevated serum sLOX-1 levels may be associated with the progression of atherosclerosis in children with T1DM. The results of this study have the potential to identify a new application area for slox-1, a biomarker detectable in serum. These potential applications are the detection of atherosclerosis in children with type-1 diabetes mellitus at a subclinical stage and monitoring of the atherosclerotic process. Consequently, further clinical studies in this area are imperative.

## Figures and Tables

**Table 1:** Demographic and Clinical Characteristics.

	Control Group (*n* = 72)	Patient Group (*n* = 80)	*p* Value
Age, years	16.3 ± 2.8	16.4 ± 2.8	0.779
Gender			
Male, *n*	39 (48.8%)	40 (55.6%)	0.402
Female, *n*	41 (51.3%)	32 (44.4%)	
BMI, kg/m^2^	20.1 ± 2.6	20.2 ± 3.2	0.897
BSA, m^2^	1.60 ± 0.19	1.61 ± 0.18	0.675
Systolic BP	114.6 ± 8.6	112.4 ± 8.5	0.280
Diastolic	76.5 ± 5.5	75.5 ± 10.0	0.190
Insulin dose, U/kg/day	-	1.0 ± 0.0	
Conventional Subcutaneous Insulin Injection, *n*	-	46 (57.5%)	
Injection per day			
1–2	-	0	
3	-	0	
≥4	-	46 (57.5%)	
Continuous Glucose Monitoring Systems (Insulin Pump), *n*	-	34 (42.5%)	
Duration of diabetes, year	-	11.0 ± 1.8	
Mean HbA1c, (%)	-	8.8 ± 1.6	
FBG, mg/dl	-	197.9 ± 87.6	-

BMI: body mass index, BSA: body surface area, BP: blood pressure, mean HbA1c: mean haemoglobin, FBG: Fasting blood glucose.

**Table 2:** Biochemistry, sLOX-1 and cIMT Measurement Results.

	Control Group (*n* = 72)	Patient Group (*n* = 80)	*p* Value
GFR, mL/min/1.73 m^2^	94.3 ± 2.9	94.4 ± 2.9	0.684
Urea, mg/dl	13.5 ± 2.2	14.0 ± 1.5	0.203
Creatinine, mg/dl	8.1 ± 0.0	8.3 ± 0.1	0.126
Uric acid, mg/dl	4.8 ± 0.5	4.5 ± 0.5	0.836
AST, IU/I	23.7 ± 5.0	24.4 ± 8.0	0.468
ALT, IU/I	18.4 ± 5.6	19.9 ± 7.1	0.479
Total Cholesterol, mg/dl	145.5 ± 22.6	166.1 ± 27.4	0.000
LDL, mg/dl	85.6 ± 27.2	91.1 ± 24.9	0.132
HDL, mg/dl	48.3 ± 9.2	58.2 ± 13.3	0.000
Triglyceride, mg/dl	94.9 ± 57.0	130.8 ± 176.6	0.284
TSH, mIU/L	2.6 ± 1.2	2.6 ± 1.3	0.755
Na^+^, mEq/L	137.5 ± 2.5	137.8 ± 2.4	0.712
K^+^, mEq/L	4.4 ± 0.3	4.4 ± 0.3	0.905
Hemoglobin, g/dL	12.9 ± 1.3	13.2 ± 1.1	0.037
RBC, 10^12^/L	4.4 ± 1.4	4.3 ± 1.0	0.092
MCH, pg/cell	29.8 ± 7.7	30.2 ± 5.0	0.251
MCHC, Hgb/cell	36.5 ± 8.2	34.05 ± 6.1	0.079
MCV, µm3	89.4 ± 5.6	91.0 ± 5.1	0.550
WBC, 10^9^/L	6.9 ± 1.9	6.7 ± 2.1	0.223
Platelet, 10^9^/L	324.0 ± 81.6	316.5 ± 65.5	0.507
hs-CRP, mg/dl	0.09 ± 0.12	0.39 ± 0.26	0.000
sLOX-1, ng/ml	0.49 ± 0.11	0.82 ± 0.35	0.000
cIMT, mm	0.35 ± 0.03	0.47 ± 0.09	0.000

GFR: glomerular filtration rate, ALT: alanine aminotransferase, AST: Aspartate, Aminotransferase, LDL: low-density lipoprotein cholesterol, HDL: high-density lipoprotein cholesterol, TSH: thyroid stimulating hormone, Na^+^: sodium ion, K^+^: potassium ion, RBC: total red blood cell count, MCV: Mean Erythrocyte Volume, MCH: Mean Erythrocyte hemoglobin, MCHC: Mean Erythrocyte Hemoglobin Concentration, hs-CRP: high sensitive C-reactive protein, cIMT: carotid intima-media thickness, sLOX-1: soluble lectin-like oxidised low-density lipoprotein receptor-1.

**Table 3:** Results of Multivariate Logistic Regression Analysis.

	Univariate Model			Multivariate Model		
	Odds Ratio	95% Confidence Interval	*p*-Value	Odds Ratio	95% Confidence Interval	*p*-Value
cIMT	1.024	1.016–1.032	0.000	1.019	1.006–1.032	0.000
Total cholesterol	1.034	1.019–1.050	0.000			
HDL	1.092	1.050–1.135	0.000	1.158	1.070–1.254	0.000
Hemoglobin	1.231	0.967–1.567	0.091			
hs-CRP	1.080	1.049–1.230	0.003	0.775	0.525–0.875	0.003
sLOX-1	1.972	1.694–2.228	0.000	1.670	1.485–1.915	0.000

cIMT: carotid intima-media thickness, HDL: high-density lipoprotein cholesterol, hs-CRP: high sensitive C-reactive protein, sLOX-1: soluble lectin-like oxidised low-density lipoprotein receptor-1.

**Table 4:** Correlation Analysis.

	sLOX-1	
	r	*p*
cIMT	0.669	0.000
hs-CRP	0.188	0.103
Mean-HgA1c	0.015	0.577
HDL	0.075	0.623

cIMT: carotid intima-media thickness, hs-CRP: high sensitive C-reactive protein, HDL: high-density lipoprotein cholesterol, Mean-HgA1c: Mean- haemoglobinA1c.

## Data Availability

The data used to support the findings of this study are available from the corresponding author upon request.

## Abbreviations

AGEs	Advanced glycation end products
ACVD	Atherosclerotic cardiovascular disease
BMI	Body mass index
cIMT	Carotid intima-media thickness
CRP	C-reactive protein
ELISA	Enzyme-linked immunosorbent assay
HDL	High density lipoprotein
hs-CRP	High sensitive C-reactive protein
ISPAD	International Society for Paediatric and Adolescent Diabetes
LDL	Low-density lipoprotein cholesterol
LOX-1	Lectin-like oxidised low-density lipoprotein receptor-1
sLOX-1	soluble Lectin-like oxidised low-density lipoprotein receptor-1
MMP-10	Metalloprotease-10
MIS-C	multisystem inflammatory syndrome in children
ox-LDL	Oxidised low-density lipoprotein
PCOS	Polycystic ovary syndrome
T1DM	Type 1 diabetes mellitus
T2DM	Type 2 diabetes mellitus
